# Correlation of predisposing factors and Esophageal Malignancy in high risk population of Baluchistan

**DOI:** 10.12669/pjms.38.3.4612

**Published:** 2022

**Authors:** Syed Muhammad Ishaque, Muhammad Sadiq Achakzai, Ziauddin, Shahid Pervez

**Affiliations:** 1 Syed Muhammad Ishaque, Associate Professor, Department of Pathology, Bolan Medical College, Sandeman Provincial/ Bolan Medical Complex Hospitals, Quetta, Pakistan; 2 Muhammad Sadiq Achakzai Associate Professor, Department of Gastroenterology, Bolan Medical College, Sandeman Provincial/ Bolan Medical Complex Hospitals, Quetta, Pakistan; 3 Ziauddin Assistant Professor, Department of Biotechnology CASVAB University of Balochistan Quetta,; 4 Shahid Pervez Professor of Pathology, Department of Pathology & Laboratory Medicine, The Agha Khan University hospital Karachi, Pakistan

**Keywords:** Cigarette, Drugs, Esophageal carcinoma (E-ca), Fruits, Fish, Risk factor, Milk, Quid, Tea, Vegetable

## Abstract

**Objectives:**

To determine frequency of esophageal malignancy in Balochistan and to evaluate its correlation with predisposing and dietary factors.

**Methods:**

This cross-sectional study was conducted from Jan 2019 to Dec 2020, at two tertiary care hospital of Quetta which caters to the entire population of province. The total number of 207 cases of esophageal biopsies were received and morphological diagnosis done by H&E staining.

**Results:**

Out of 207 (N) esophageal biopsies cases, malignancy were observed in 65%, chronic esophagitis in 19%, benign esophageal lesion in 1% and other esophageal lesions were observed in less than 4% of samples. Association with aggravating factors included tea 80.5%, use of drugs 64%, spicy food 57%, salted food 53%, quid & tobacco taken orally and through nose 44% and cigarette smoking 21.5%. The protective factors include fresh fruit 90%, fish 64% and milk 55% which were never or occasionally taken, meat chicken and beef intake was 63% & 53% and vegetable intake was 52%, but 72.5% of cases never used alcohol and mutton meat was not used by 50%.

**Conclusion:**

Esophageal cancer was associated in those groups of people which may regard as having high risk factors. These include increased intake of spicy/salted food, hot beverages, drugs, quid and smoked tobacco, coupled with low intake of fruits and vegetables, lack of awareness and low socioeconomic status.

## INTRODUCTION

Esophageal malignancy is a highly virulent cancer with poor survival/prognosis and five years survival was hardly15% to 25%.^1^,^2^ Approximately half of esophageal cancer patients presented with distant metastasis when they were first diagnosed.^3^-^5^

Globally esophageal malignancy account for more than 400,000 deaths annually with the majority in South & East Africa and Asia.^1^,^5^ The highest incidence of esophageal cancer is greater than 100/100,000 population with the Asian esophageal cancer belt extending from North Iran through central Asian republics to North-Central China.^2^

Esophageal cancer is the 4^th^ commonly diagnosed malignancy and 4^th^ leading cause of death in China which accounted for 2,59,335 new cases and 2, 11, 084 deaths in 2008. In India esophageal malignancy represents the 3^rd^ & 4^th^ leading malignancy in males and females respectively.^6^-^8^

Globally the Quetta region of Balochistan was considered to be a high incidence region for esophageal cancer although this figures as compared to Karachi had a moderate incidence.^9^ Esophageal cancer was the seventh and sixth most common malignancy in male, female gender respectively in the Karachi region, while it’s considered the third commonest neoplasm in the Quetta region.^2^

Environmental factors, including tobacco smoking, drinking of alcohol and other dietary factors have been thought to be associated and play important carcinogenic role in esophageal malignancy.^1^,^6^,^10^ Low intake of raw vegetables, fresh fruits and deficiency of vitamins with protective anti-oxidant, intake of hot beverages leading to > thermal injuries’ have been inversely related with esophageal malignancy.^11^ Other risk factors are spicy food, red meat, poor oral hygiene, life style, behavior, socioeconomic status, medical factors such as drugs, genetics/familial cancers and level of education are also considered as contributory factors for esophageal malignancy.^12^

The aim of this study was to correlate the frequency of different esophageal lesions in population of Balochistan and to observe the various addictive and risk factors in addition to protective factors and their association with E-ca.

## METHODS

This cross sectional study was carried out at the department of Pathology/Gastroenterology Bolan Medical College Quetta, from1^st^ January 2019 to 31^st^ December 2020. A total number of 207 esophageal biopsies specimens’ were included. These endoscopic biopsies were done in department of Gasteroentology Bolan Medical Complex and Sandeman Provincial hospitals Quetta.

The surgical pathology records, relevant details like clinical presentation, history of addiction and dietary details were collected in separate proforma in all suspected malignant esophageal cases. Formalin fixed, paraffin embedded blocks was taken, thin slices of five microns were made and stained with Haematoxylin & Eosin. All slides were studied by light microscope with 04x, 10x and 40x power by two different histopathologist.

Degree of salty/spicy food, drugs, hot beverages, fruits, fish, milk, meat, tobacco and vegetables’ are categorized as mild, moderate & high risk, many, few and moderate > modified as 1+, 2 ++ & 3+++ [3,11, 13].

The inclusion criteria were those patients who presented with history of dysphagia, retro-sternal burning pain, vomiting and clinically suspected as esophageal malignancy. Exclusion criteria were those patients whose presentations did not primarily originate from esophageal lesions; poorly fixed, inadequate tissue and metastatic tumors were excluded.

Descriptive statistics were calculated in percentage and Excel was used in compiling the results.

### Ethical Committee Approval

(Ref: No.2-5/Edn;/14915/16, Dated: 24 October 2019).

## RESULTS

Our observation showed that majority of the esophageal lesions was neoplastic variants, out of 207 (N) biopsy proven cases, the frequency of E-ca was 65% followed by chronic esophagitis i-e 19% and benign esophageal lesions was only one%, while other lesions were noted four % of cases in each. (Table-I).

**Table I:** Distribution of esophageal biopsies proven lesions, (N=207).

S. No.	Esophageal lesions	Numbers (Percentage %)
01	Chronic esophagitis	39 (19%)
02	Esophagitis (other variants)	06 (03%)
03	Barrett esophagus	08 (04%)
04	Hyperplasia	08 (04%)
05	High-grade dysplasia	09 (04%)
06	Benign lesions	02 (01%)
07	Malignant lesions	135 (65%)

Total		207

Hot beverages in the form of tea & soups, drugs and spicy/salted foods are considered as major risk factors in our populations. These results are elaborated in Table-II which showed the distribution of various addictive & risk factors and its association with esophageal cancer. Out of that commonest was tea which showed 80.5% positivity, followed by drugs, spicy/salted food, quid and smoking which had 64%, 57%, 53%, 44% and 21.5% positivity respectively. Our result also showed that alcohol consumption was absent in 72.5% of cases and 27.5% cases had occasional intake with no significant correlation.

**Table II:** Distribution of various addictive and risk factors in esophageal malignancy, (N=135).

S. No.	Addictive & risk factors	Quantity				

		Negative	1+	2+	3+	Total
01	Alcohol	98 (72.5%)	37 (27.5%)	00	00	135
02	Drugs	16 (12%)	33 (24%)	70 (52%)	16 (12%)	135
03	Quid	28 (21%)	47 (35%)	45 (33%)	15 (11%)	135
04	Smoking	49 (36%)	57 (42%)	29 (21.5%)	00	135
05	Salted foods	29 (21%)	35 (26%)	63 (47%)	08 (06%)	135
06	Spicy foods	26 (19%)	32 (24%)	66 (49%)	11 (08%)	135
07	Tea	08 (06%)	18 (13%)	53 (39%)	56 (41 5%)	135

Alcohol >1+ (off & on use in a week). **Drugs** (Analgesic, proton pump inhibitor & others) >1+ (occasionally one type of drug taken daily), 2+ (two types daily), 3+ (three or more types daily). **Quid (Naswa**r) (Tobacco by mouth or by nose) > 1+ (5 time a day), 2+ (6 to10 time a day), 3+ (more than 15 times a day). **Smoking** Cigarette > 1+ (5 to 8 cigarettes/ day), 2+ (10 to 15/day), 3+ (20 or mor e/day). **Salted Food** > 1+ (taken 2 times a week), 2+ (taken once a day), 3+ (2 times a days). **Spicy Food** > 1+ (taken 2 times a week), 2+ (taken once a day), 3+ (2 times a day). **Tea** (Hot beverages) > 1+ (700 ml /day), 2+ ( up to1200 ml/day), 3+ (1700 ml or more /day).

The distribution of protective factors and its association with E-ca are listed in Table-III. Out of that 90% of lesion affected people may never or occasionally use fresh fruits, while 64% never use fish, 55% occasionally use milk. The intake of various type of meat like chicken, beef & mutton showed that 63% & 53% and only 04% were taking four or more times in a week, while mutton, beef and chicken intake was rare in 50% 19% and 12 % of cases. The consumption of vegetable was seen in 52% of cases, which may take 4-5 times in a week but these were in very low quantity.

**Table III:** Distribution of various protective factor and esophageal malignancy, (N=135).

S. No.	Protective items	Quantity				

		Negative	1+	2+	3+	Total
01	Fresh Fruits	33 (24%)	89 (66%)	13 (10%)	00	135
02	Fish	86 (64%)	45 (33%)	04 (03%)	00	135
03	Milk	56 (41%)	74 (55%)	05 (04%)	00	135
04	Beef	26 (19%)	38 (28%)	66 (49%)	05 (04%)	135
05	Chicken	16 (12%)	34 (25%)	75 (55.5%)	10 (7.5%)	135
06	Mutton	68 (50%)	62 (46%)	05 (04%)	00	135
07	Vegetables	09 (07%)	55 (41%)	68 (50%)	03 (02%)	135

**Fresh Fruits** > 1+ (2 time a week), 2+ (3-4 time a week), 3+ (more than 5 times a week). **Fish** >1+ (off & on, occasionally in a week), 2+ (2 time a week), 3+ (5 time a week). **Milk** > 1+ (300 ml or more in a week), 2+ (500 ml on every 3^rd^ day), 3+ (more than 1000 ml in a week).** Beef** > 1+ (2 times a week), 2+ (3-4 times a week), 3+ (more than 5 times a week). **Chicken** > 1+ (2 times a week), 2+ (3-4 times a week), 3+ (more than 5 times a week). **Mutton** > 1+ (2 times a week), 2+ (3-4 times a week), 3+ (more than 5 times a week). **Vegetables** > 1+ (2 times a week), 2+ (3-4 times a week) & 3+ (more than 5 times a week).

## DISCUSSION

In the present study different histopathological esophageal lesions showed that frequency of esophageal cancer is very high in our community i-e 65, 19% with chronic esophagitis and 1% with benign lesions. These results are comparable with other Pakistani studies which reported that the Quetta region has a high incidence of esophageal cancer and it is considered the third commonest neoplasm.^2^,^9^

Our study showed that use of spicy food had 57% & salted food 53% positivity. These are in comparable with other studies which reported that the major risk factors were excess intake of processed, spicy & salted foods, which have a positive and significant correlation with esophageal malignancy.^14^,^15^

The use of quid had 44% positivity and most of them were taking it by mouth but some of them through nose and more than 12 times in a day for long time, which may also badly affect the oral hygiene. Our results also showed cigarette smoking had 21.5% positive association in these patients. Alcohol consumption is very low in our community which may be due to religious reasons, non-availability of beer and cultural habits. Mostly i-e 72.5% of cases was noted to be negative and only 27.5% cases had history of occasionally alcohol intake which may show no significant correlation with esophageal carcinoma.

Our results were in agreement with the study which reported smoking, chewing pan, eating naswar and inhaling snuff are considered as risk factors in E-ca and they also claimed that for smokers, naswar users the risk of the lesion was two and 3.3 times higher than in non-user.^16^ Other studies have observed that tobacco smoking, nass chewing and poor oral hygiene are considered as major risk factors. They further showed that in low risk areas alcohol and tobacco smoking were considered major risk factors, whereas in high risk areas its etiology was less clear and there was no significant correlation.^17^

Iranian & US studies have reported high prevalence of esophageal squamous cell carcinoma (ESCC) in Iran & China which showed smoking and alcohol were considered as minor risk factors. Other studies have reported up to 90% cases of E-ca in Westerns countries which were associated with smoking and alcohol use, whereas in the high risk areas of the Asian belt and Iran there is no significant role to consider it as a risk factors.^18^-^20^

In this study hot beverages in the form of green tea (kawa) and bread with hot soup had 80.5% positivity which led to thermal injuries of lining epithelium (mucosa), it leads to chronic inflammatory process thus leading to non-healing tears/ulcers > dysplasia and ESCC. Almost similar findings were observed by groups of researchers who reported that hot beverages’ in high quantities may be considered as risk factors. A study from Ludhiana Punjab reported that use of hot beverages, malnourished and low socioeconomic status were considered as major risk factors for ESCC.^15^,^17^

The University of Georgia reported that tea in the form of green, black or colong contains the active component polyphenol which may lead to ESCC. Some researchers have reported that high temperature tea intake was associated with 1.6 fold increase risk of E-ca in Japanese, and also showed 32 of 47 independent reports from 39 different studies to have a strong correlation between hot beverages and ESCC.^7^,^21^,^22^

Our study shows that 64% of the E-ca affected patients used different types of drugs i-e pain killer, proton pump inhibitors and others, due to illiteracy, initially use self-medication, than by relative suggestions, in 3^rd^ step from medical store & general practitioners and lastly came to consultant opinions which were so late. These results compared with the study who reported that different chemicals, drugs and opium intakes were considered as risk factors for E-ca.^17^

Our study also showed that occasional intake of fresh fruit, milk was 66%, 55% & vegetable intake had 52% positivity, whereas fish intake was absent in 64% of cases. Similar finding was reported that if fruit and vegetable intake increases to 100 gram/day this can decrease the incidence of E-ca up to 11%. Another study reported low fruits and vegetables’ consumption have an inverse association with ESCC. Brazilian study showed that consumption of vegetable, fresh fruits and juices were considered as protective factors for esophageal malignancy.^7^,^14^,^23^

In this study the meat either in form of chicken or beef had 63% & 53%positivity, whereas mutton intake was only 4% all of them used in very low quantity. Red meat contains increased amount of iron (heme) which may act as pro-oxidation and able to catalyze lipid per oxidation with DNA damage at the cellular level. Our results are in agreement with studies which reported that high consumption of red meat, processed meat and barbequed meat were associated with increased E-ca, whereas white meat (chicken and fish) were found to have protective effects.^24^,^25^ Other studies have reported positive correlation between red meat intake and ESCC. They claimed that increased intake of red meat either processed or in barbecued forms have strong association and are considered as risk factor for esophageal malignancy.^26^

## CONCLUSION

E-ca has positive correlations with various dietary & addictive factors, low intake of fruits & vegetables, lack of awareness and low socioeconomic status. As such health education to the community and health care provider to change the life styles to decrease the spread of said lesions. As esophageal malignancy is high in frequency, so those patients who present with esophageal complaints. endoscopic biopsies’ are recommended after every 9-12 months for early diagnosis and timely management.

### Authors’ Contribution:

**SMI, ZN & SP:** Conceived/design, analysis and editing of manuscript and agreement to be accountable.

**MSA:** Help in data collection, drafting the manuscript and statistical analysis.
